# Transetherification on Polyols by Intra- and Intermolecular Nucleophilic Substitutions

**DOI:** 10.1371/journal.pone.0091912

**Published:** 2014-03-24

**Authors:** Takahiro Muraoka, Kota Adachi, Rainy Chowdhury, Kazushi Kinbara

**Affiliations:** 1 Institute of Multidisciplinary Research for Advanced Materials, Tohoku University, Aoba-ku, Sendai, Japan; 2 PRESTO, Japan Science and Technology Agency, Kawaguchi, Saitama, Japan; University of Melbourne, Australia

## Abstract

Transetherification on polyols involving intra- and intermolecular nucleophilic substitutions is reported. Di- or trialkoxide formation of propane-1,3-diol or 2-(hydroxymethyl)propane-1,3-diol derivatives by NaH triggers the reaction via oxetanes formation, where the order to add NaH and a polyol significantly influences the yields of products. It was demonstrated that the protective group on the pentaerythritol skeleton is apparently transferred to the hydrophilic and hydrophobic chain molecules bearing a leaving group in one-step, and a protective group conversion from tosyl to benzyl was successful using a benzyl-appending triol to afford a desired product in 67% yield.

## Introduction

An ether synthesis is one of key reactions in preparation of materials including long hydrophilic or hydrophobic tails [1]–[10]. Usually, an alkoxy anion, generated by the hydrogen abstraction from an alcohol with a strong base, reacts with the target long chain molecule bearing a leaving group, like tosyl and halide moieties. This methodology is also applicable for preparation of branched molecules bearing multiple chains like dendrimers, amphiphiles, or liquid crystalline molecules, where a polyol, such as pentaerythritol, provides one of the fundamental skeletons to construct such branched structures [11]–[22]. Transetherification is also a useful reaction for the ether synthesis to develop functional molecules and hyperbranched polymers [23]–[32]. However, transetherification can also be an adverse side reaction in a multi-step reaction scheme [33]–[35]. Here we report our serendipitous discovery of transetherification, which proceeds by intra- and intermolecular nucleophilic substitutions starting from protected pentaerythritols coupled with chain molecules bearing a leaving group. This reaction scheme would offer a possible route for preparation of ethers and also predict a side reaction in the synthesis of branched compounds.

## Results and Discussion

**Figure 1:**
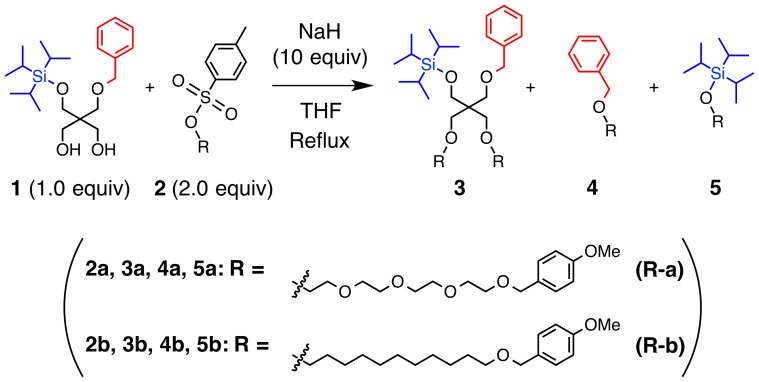
Ether formation between 1 and 2.

In our research project to develop structured poly(ethylene glycols) [36], we tried Williamson ether synthesis [37] between a propane-1,3-diol derivative **1** and a tosylate **2a** with NaH in tetrahydrofuran (THF; Figure 1, Table 1, Entry 1). Initially **1** was mixed with NaH in anhydrous THF, and the mixture was heated under reflux for generation of the alkoxide. The resulting mixture gave a deep red solution, where **2a** was added at 0°C (Procedure A). Actually, this reaction afforded the expected product **3a** in 13% yield. Meanwhile, **4a** (21% yield) was unexpectedly obtained as the major product with a comparable amount of **5a** (12%). Apparently, transetherification of benzyl and triisopropylsilyl (TIPS) groups of **1** to **2a** took place by substitution with the tosyl group, together with the formation of the ether linkage at the hydroxy group of **1** to give **3**. A product due to one-to-one coupling between **1** and **2a** was not detected. Such unexpected products were obtained not only with the oligoethylene glycol tosylate, but also with tosylate **2b** having a hydrophobic alkyl chain, where the reaction under similar condition resulted in the formation of **4b** and **5b** in 21% and 6% yield, respectively, in addition to **3b** (Table 1, Entry 2).

**Table 1:** Ether formation between 1 and 2.

Entry	R	Procedure	[1] (mM)	[2] (mM)	Yields (% vs. 1)^c)^		
					3	4	5
1a)	R-a	A	32.5	65.0	13	21	12
2a)	R-b	A	32.5	65.0	33	21	6
3a)	R-a	B	32.5	65.0	93	2	1
4^b)^	R-a	A	16.5	33.0	37	10	9

a) Reaction conditions: 30 mL THF, 0.972 mmol **1**, 1.94 mmol **2**, 9.72 mmol NaH; reflux (ca. 339 K); reaction time: 12 h. ^b)^ Reaction conditions: 30 mL THF, 0.486 mmol **1**, 0.972 mmol **2**, 4.86 mmol NaH; reflux (ca. 339 K); reaction time: 12 h. ^c)^ Isolated yields.

**Figure 2:**
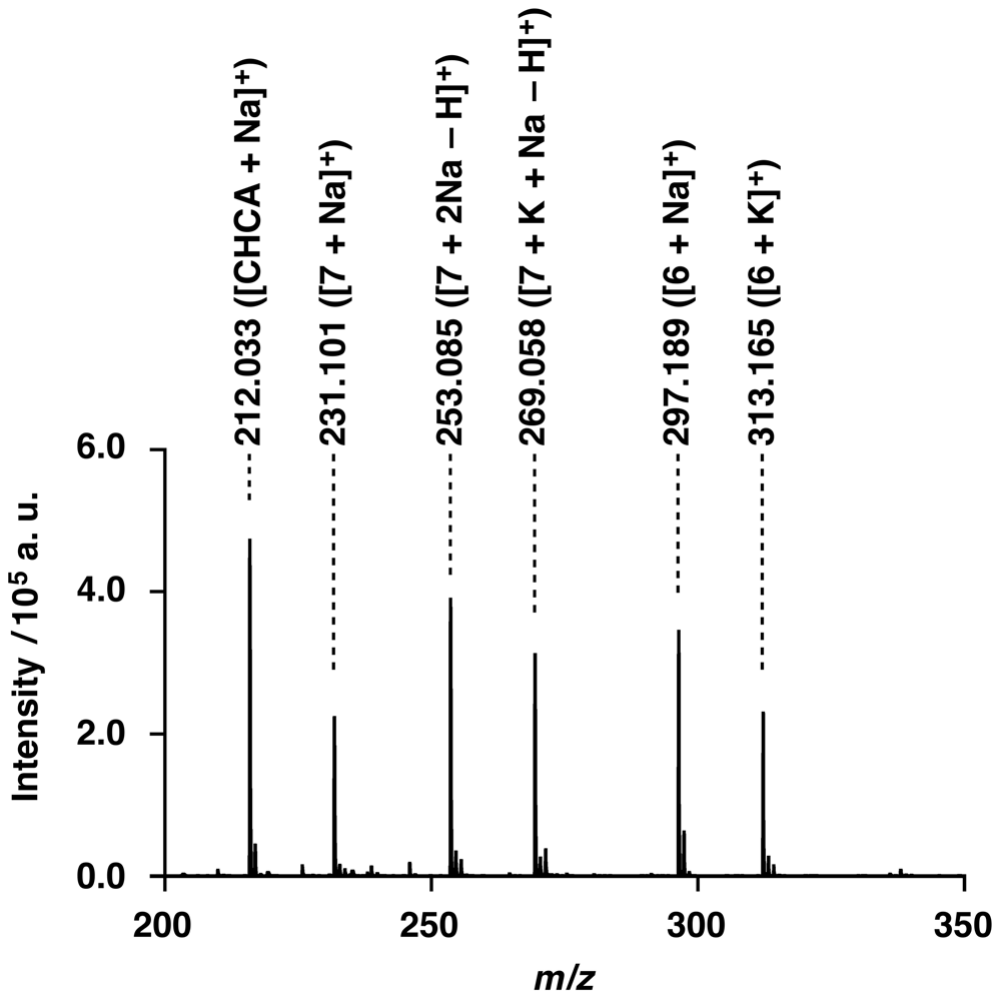
MALDI-TOF-MS spectrum of the crude product extracted by CHCl_3_ for the reaction in Table 1, Entry 1. Structures of 6 and 7 are shown in Figure 3. Matrix: α-cyano-4-hydroxycinnamic acid.

**Figure 3:**
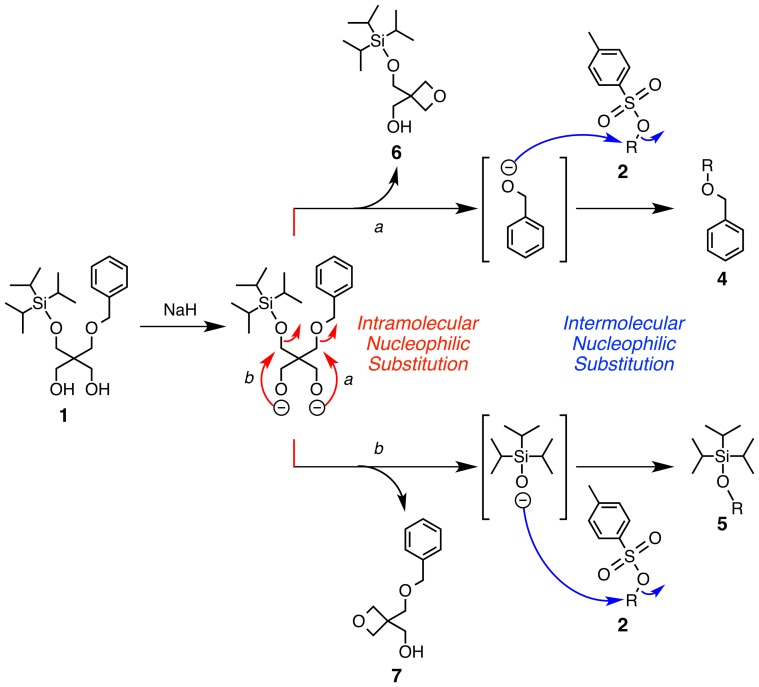
A plausible reaction mechanism of the intra- and intermolecular nucleophilic substitutions to prompt transetherification.

Here, it is of importance that, the MALDI-TOF-MS spectrum of the crude product with α-cyano-4-hydroxycinnamic acid as a matrix (Figure 2), extracted with CHCl_3_ from the reaction mixture (Table 1, Entry 1), shows molecular ion peaks corresponding to oxetane derivatives **6** and **7** (Figure 3) (Calcd for C_12_H_16_NaO_3_: 231.0997 ([**7**+Na]^+^), C_12_H_15_Na_2_O_3_: 253.0817 ([**7**+2Na – H]^+^), C_12_H_15_KNaO_3_: 269.0556 ([**7**+Na+K – H]^+^), C_14_H_30_NaO_3_Si: 297.1862 ([**6**+Na]^+^) and C_14_H_30_KO_3_Si: 313.1601 ([**6**+K]^+^)). The MALDI-TOF-MS spectrum of the crude product with gentisic acid as a matrix also showed molecular ion peaks corresponding to oxetane derivatives **6** and **7** (Found: 231.616 ([**7**+Na]^+^), 253.397 ([**7**+2Na – H]^+^), 269.230 ([**7**+Na+K – H]^+^) and 297.097 ([**6**+Na]^+^)). Yields of **6** and **7**, evaluated by ^1^H-NMR spectroscopy, were 20% and 12%, respectively, which almost correspond to the yields of **4a** (21%) and **5a** (12%). This result suggests that the alkoxide of **1** formed by the reaction with NaH undergoes an intramolecular nucleophilic substitution to form oxetanes **6** or **7**. This likely accompanies the formation of nucleophilic benzyloxy or siloxy anions, which finally react with **2a** to yield **4a** or **5a**, respectively.

**Figure 4:**
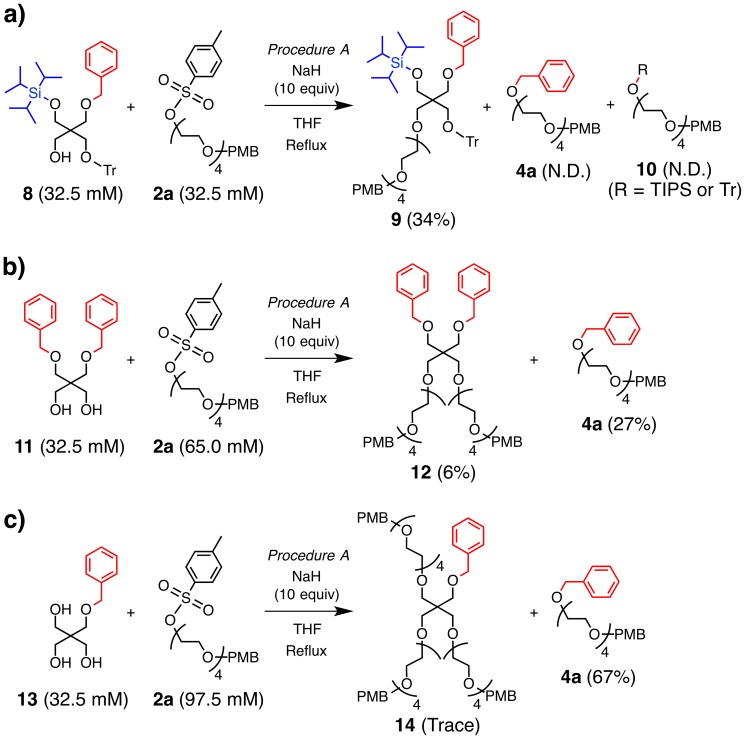
Ether formation between tetraethylene glycol tosylate 2a and a) monoalcohol 8, b) propane-1,3-diol 11 and c) 2-(hydroxymethyl)propane-1,3-diol 13. Reaction time was 12 h. Yields were calculated based on the isolated amounts. ND: not detected.

Noteworthy here is that the order of the addition of reagents, namely that of NaH, **2a** and **1**, significantly influenced on the yields of the products. When NaH was added to the mixture of **1** and **2a**, followed by refluxing (Procedure B), **3a** was obtained in 93% yield, while the formation of **4a** and **5a** was negligible (Table 1, Entry 3). Under this condition, the reaction mixture remained colorless, unlike Procedure A, indicating formation of monoalkoxide of **1**. Furthermore, when the reaction was carried out with a half concentration of **1**, **2** and NaH in Procedure A (Table 1, Entry 4), the yield of **3a** was increased (37%), while yields of **4a** and **5a** were decreased (10% and 9% yield, respectively). The dilute condition is likely favorable for the formation of the monoalkoxide of **1**. Hence, these results suggest that the suppression of dialkoxide formation from **1** would be advantageous for the formation of **3a**, while being disadvantageous for the formation of **4a** and **5a**. Indeed, a reaction between monoalcohol **8** and **2a** with NaH, following Procedure A, afforded **9** in 34% yield, while **4a**, **5a**, and **10** were not detected (Figure 4a). Thus, the intra- and intermolecular nucleophilic substitutions to prompt the transetherification are likely triggered by a dianion formation from **1**.

A tosyl group functions as a protecting group for alcohols [38],[39]. Hence, this transetherification can be regarded as a one-step method to convert the protecting group from tosyl to another one such as benzyl or TIPS. To demonstrate the protecting group conversion from tosyl to benzyl, 2,2-bis((benzyloxy)methyl)propane-1,3-diol **11** and 2-((benzyloxy)methyl)-2-(hydroxymethyl)propane-1,3-diol **13** were reacted with tosylate **2a** (Figures 4b and 4c). A reaction between **11** and **2a** with NaH in THF following Procedure A afforded **4a** in 27% yield with the formation of **12** in 6% yield. Importantly, a reaction between **13** and **2a** resulted in the formation of **4a** in much higher yield (67%), with a trace amount of **14**. Products due to one-to-one and one-to-two coupling between **13** and **2a** were not detected. The neighboring three hydroxy groups in **13** are likely advantageous for the formation of dialkoxide or trialkoxide to encourage the transetherification. Thus, the triol **13** is a useful reagent for the protecting group transfer to the tosyl group through the transetherification by intra- and intermolecular nucleophilic substitutions.

In this work, transetherification of polyols involving intra- and intermolecular reactions was reported. It is strongly likely that the di- or trialkoxide formation triggers the transetherification. These results are considered not only to lead to new synthetic routes for preparing ethers and branched compounds, but also to be useful to avoid adverse side reactions related to Williamson ether synthesis [40]–[44]. Using this reaction, one-step transfer of a hydroxy-protecting group from benzyl to tosyl was also successfully demonstrated.

## Experimental Part

### General

Column chromatography: with silica gel (SiO_2_; 63–210 μm; Kanto Chemical). ^1^H-NMR spectra: Bruker BioSpin AVANCE III 400 and BioSpin AVANCE III 500 FT-NMR spectrometers; in CDCl_3_; *δ* in ppm rel. to Me_4_Si as an internal standard, *J* in Hz. where the chemical shifts were determined with respect to Me_4_Si as an internal standard. MALDI-TOF-MS spectra (pos. ref. mode): Bruker Daltonics autoflex speed spectrometer; α-cyano-4-hydroxycinnamic acid and gentisic acid as a matrix. HR-ESI-TOF-MS spectra (pos. mode): Bruker Daltonics micrOTOF-Q II spectrometer.

### Ether Formation

Procedure A: A mixture of **1** (0.372 g, 0.972 mmol) and NaH (0.233 g, 9.72 mmol) in anhydrous THF (15 mL) was refluxed (about 339 K) under Ar for 30 min in the dark, where the reaction mixture turned into deep red from a colorless suspension. After the mixture was cooled to 273 K, an anhydrous THF solution (15 mL) of **2a** (0.911 g, 1.94 mmol) was added dropwise to the resulting mixture. After the reaction mixture was refluxed for 12 h in the dark, water (50 mL) was added to the resulting mixture at 0°C, and organic components were extracted with CHCl_3_ (3×50 mL). The organic extract was dried over Na_2_SO_4_ and filtered off from insoluble substances. The filtrate was evaporated to dryness under reduced pressure at 313 K, and the residue was purified by column chromatography (EtOAc/hexanes/MeOH 90∶10∶0 to 100∶0∶0 to 90∶0∶10) to afford **1** (recovered, 0.134 g, 0.350 mmol, 36%), **3a** (0.123 g, 0.126 mmol, 13%), **4a** (0.083 g, 0.204 mmol, 21%), and **5a** (0.055 g, 0.117 mmol, 12%).

Procedure B: To an anhydrous THF (30 mL) solution of **1** (0.371 g, 0.972 mmol) and **2a** (0.909 g, 1.94 mmol) was added NaH (0.234 g, 9.72 mmol) at 0°C under Ar. After the reaction mixture was refluxed (about 339 K) for 12 h in the dark, water (50 mL) was added to the resulting mixture at 273 K, and organic components were extracted with CHCl_3_ (3×50 mL). The organic extract was dried over Na_2_SO_4_ and filtered off from insoluble substances. The filtrate was evaporated to dryness under reduced pressure at 313 K, and the residue was purified by column chromatography (EtOAc/hexanes/MeOH 90∶10∶0 to 100∶0∶0 to 90∶0∶10) to afford **1** (recovered, 0.007 g, 2%), **3a** (0.882 g, 0.904 mmol, 93%), **4a** (0.008 g, 0.019 mmol, 2%), and **5a** (0.005 g, 0.0097 mmol, 1%).

For characterization of **1**, **2a**, **3a**, **8** and **13**, see [36].

Data of **2b**: ^1^H-NMR: 1.21–1.35 (*m*, 14H); 1.56–1.64 (*m*, 4H); 2.45 (*s*, 3H); 3.43 (*t*, *J* = 6.5, 2H); 3.80 (*s*, 3H); 4.02 (*t*, *J* = 6.5, 2H); 4.43 (*s*, 2H); 6.88 (*d*, *J* = 8.0, 2H); 7.27 (*d*, *J* = 7.0, 2H); 7.34 (*d*, *J* = 8.0, 2H); 7.79 (*d*, *J* = 7.0, 2H). MALDI-TOF-MS: 485.30 ([*M*+Na]^+^, C_26_H_38_NaO_5_S^+^; calc. 485.23).

Data of **3b**: ^1^H-NMR: 1.02–1.08 (*m*, 18H); 1.26–1.35 (*m*, 28H); 1.51 (*m*, 3H); 1.56–1.61 (*m*, 8H); 3.33–3.46 (*m*, 16H); 3.80 (*s*, 6H); 4.43 (*s*, 4H); 4.48 (*s*, 2H); 6.87 (*d*, *J* = 8.5, 4H); 7.25–7.31 (*m*, 9H). HR-ESI-TOF-MS: 985.6926 ([*M*+Na]^+^, C_59_H_98_NaO_8_Si^+^; calc. 985.6929).

Data of **4a**: ^1^H-NMR: 3.58–3.68 (*m*, 16H); 3.80 (*s*, 3H,); 4.49 (*s*, 2H); 4.56 (*s*, 2H); 6.87 (*d*, *J* = 8.5, 2H); 7.27 (*m*, 4H); 7.33 (*m*, 3H). HR-ESI-TOF-MS: 427.2098 ([*M*+Na]^+^, C_23_H_32_NaO_6_^+^; calc. 427.2097).

Data of **4b**: ^1^H-NMR: 1.07–1.36 (*m*, 14H); 1.57–1.63 (*m*, 4H); 3.36–3.48 (*m*, 4H); 3.80 (*s*, 3H); 4.43 (*s*, 2H); 4.51 (*s*, 2H); 6.88 (*d*, *J* = 8.5, 2H); 7.26 (*d*, *J* = 8.5, 2H); 7.27–7.31 (*m*, 5H). HR-ESI-TOF-MS: 421.2718 ([*M*+Na]^+^, C_26_H_38_NaO_3_^+^; calc. 421.2719).

Data of **5a**: ^1^H-NMR: 1.02–1.11 (*m*, 21H); 3.56–3.68 (*m*, 14H); 3.80 (*s*, 3H); 3.83 (*t*, *J* = 5.5, 2H); 4.49 (*s*, 2H); 6.87 (*d*, *J* = 8.5, 2H); 7.26 (*d*, *J* = 8.5, 2H). HR-ESI-TOF-MS: 493.2965 ([*M*+Na]^+^, C_25_H_46_NaO_6_Si^+^; calc. 493.2961); 509.2704 ([*M*+K]^+^, C_25_H_46_KO_6_Si^+^; calc. 509.2701).

Data of **5b**: ^1^H-NMR: 1.03–1.08 (*m*, 18H); 1.26–1.35 (*m*, 16H); 1.55–1.59 (*m*, 5H); 3.43 (*t*, *J* = 7.0, 2H); 3.75 (*t*, *J* = 7.0, 2H); 3.80 (*s*, 3H); 4.43 (*s*, 2H); 4.48 (*s*, 2H); 6.87 (*d*, *J* = 8.5, 2H); 7.29 (*d*, *J* = 8.5, 2H). HR-ESI-TOF-MS: 487.3585 ([*M*+Na]^+^, C_28_H_52_NaO_3_Si^+^; calc. 487.3583).

Data of **6**: ^1^H-NMR: 1.04 (*s*, 6H); 1.05 (*s*, 12H); 1.57 (*m*, 3H); 3.70 (*s*, 2H); 3.94 (*s*, 2H); 4.45 (*d*, *J* = 6.0, 2H); 4.48 (*d*, *J* = 6.0, 2H). MALDI-TOF-MS: 297.189 ([*M*+Na]^+^, C_14_H_30_NaO_3_Si^+^; calc. 297.186); 313.165 ([*M*+K]^+^, C_14_H_30_KO_3_Si^+^; calc. 313.160).

Data of **7**: ^1^H-NMR: 3.70 (*s*, 2H); 3.95 (*s*, 2H); 4.45 (*d*, *J* = 6.0, 2H); 4.49 (*d*, *J* = 6.0, 2H); 4.54 (*s*, 2H); 7.29–7.35 (*m*, 5H). MALDI-TOF-MS: 231.101 ([*M*+Na]^+^, C_12_H_16_NaO_3_^+^; calc. 231.100).

Data of **9**: ^1^H-NMR: 0.92–1.01 (*m*, 21H); 3.16–3.69 (*m*, 16H); 3.55 (*s*, 6H); 3.63 (*s*, 2H); 4.43 (*s*, 2H); 4.47 (*s*, 2H); 6.85 (*d*, *J* = 8.5, 2H); 7.17–7.27 (*m*, 20H); 7.40 (*d*, *J* = 8.0, 2H). HR-ESI-TOF-MS: 943.5151 ([*M*+Na]^+^, C_56_H_76_NaO_9_Si^+^; calc. 943.5156); 959.4890 ([*M*+K]^+^, C_56_H_76_KO_9_Si^+^; calc. 959.4896).

Data of **11**: ^1^H-NMR: 2.59 (*t*, *J* = 6.0, 2H); 3.57 (*s*, 4H); 3.69 (*s*, 4H); 4.50 (*s*, 4H); 7.26–7.33 (*m*, 10H). HR-ESI-TOF-MS: 339.1576 ([*M*+Na]^+^, C_19_H_24_NaO_4_^+^; calc. 339.1572).

Data of **12**: ^1^H-NMR: 3.54–3.68 (*m*, 40H); 3.80 (*s*, 3H); 4.49 (*s*, 2H); 4.50 (*s*, 2H); 6.87 (*d*, *J* = 8.5, 2H); 7.26–7.30 (*m*, 14H). MALDI-TOF-MS: 931.46 ([*M*+Na]^+^, C_51_H_72_NaO_14_^+^; calc. 931.48); 947.43 ([*M*+K]^+^, C_51_H_72_NaO_14_^+^; calc. 947.45).

Data of **14**: ^1^H-NMR: 3.37–3.67 (m, 48H); 3.793 (s, 6H); 3.802 (s, 3H); 4.46 (s, 2H); 4.485 (s, 4H); 4.494 (s, 2H); 6.86–6.88 (m, 6H); 7.25–7.31 (m, 14H). HR-ESI-TOF-MS: 1153.5716 ([*M*+K]^+^, C_60_H_90_KO_19_^+^; calc. 1153.5713).
